# Exploring the Potential of Micro-Immunotherapy in the Treatment of Periodontitis

**DOI:** 10.3390/life14050552

**Published:** 2024-04-25

**Authors:** Maria del Mar Ferrà-Cañellas, Laura Garcia-Sureda

**Affiliations:** 1 Preclinical Research Department, Labo’Life España, 07330 Consell, Spain; 2 Group of Cell Therapy and Tissue Engineering, Research Institute on Health Sciences (IUNICS), University of the Balearic Islands, 07122 Palma de Mallorca, Spain; 3 Health Research Institute of the Balearic Islands (IdISBa), 07122 Palma de Mallorca, Spain

**Keywords:** periodontitis, micro-immunotherapy, low doses, ultra-low doses, immunotherapy, immunomodulation, cytokines

## Abstract

Periodontitis, characterized by the progressive destruction of dental support tissues due to altered immune responses, poses a significant concern for public health. This condition involves intricate interactions between the immune response and oral microbiome, where innate and adaptive immune responses, with their diverse cell populations and inflammatory mediators, play crucial roles in this immunopathology. Indeed, cytokines, chemokines, growth factors, and immune cells perform key functions in tissue remodeling. Focusing on periodontal therapies, our attention turns to micro-immunotherapy (MI), employing low doses (LDs) and ultra-low doses (ULDs) of immunological signaling molecules like cytokines, growth factors, and hormones. Existing studies across various fields lay the groundwork for the application of MI in periodontitis, highlighting its anti-inflammatory and regenerative potential in soft tissue models based on in vitro research. In summary, this review underscores the versatility and potential of MI in managing periodontal health, urging further investigations to solidify its clinical integration. MI supports an innovative approach by modulating immune responses at low doses to address periodontitis.

## 1. Introduction

Periodontal diseases are characterized by the progressive destruction of tissues that support teeth, including the soft and hard tissues of the periodontal region [1]. These conditions, which pose a significant challenge to oral and public health, result from an altered immune response and the disruption of the oral ecosystem, where the microbial community plays a crucial role [2,3,4].

Periodontal diseases, such as gingivitis and periodontitis, arise from the complex interaction between the immune response and microbial biofilms in the periodontal region. Gingivitis is an acute and reversible inflammation confined to the gingival tissues. If left untreated, gingivitis leads to periodontitis, a more advanced, irreversible, and destructive form of periodontal disease [1]. Periodontal disease represents a significant public health problem that affects 20–50% of the adult population, with chronic periodontitis being the most prevalent form [4,5,6,7]. This context underscores the importance of understanding the mechanisms involved in the development and progression of the disease.

Gingivitis is caused by pathogenic subgingival microbial biofilm development and dysbiotic interactions between the host and the hosted microbes. The extracellular matrix on the teeth surface at the initial stage of biofilm formation facilitates this process, leading to anaerobic microorganisms increasing due to oxygen depletion in a growing and mature biofilm [8]. This transition allows the dysbiotic community, rich in virulence factors, to prosper in an inflammatory environment [9]. Consequently, pathogenicity occurs when the host’s immune response is dysregulated due to changes in the microbial community or immune-regulatory defects [10].

**Figure 1:**
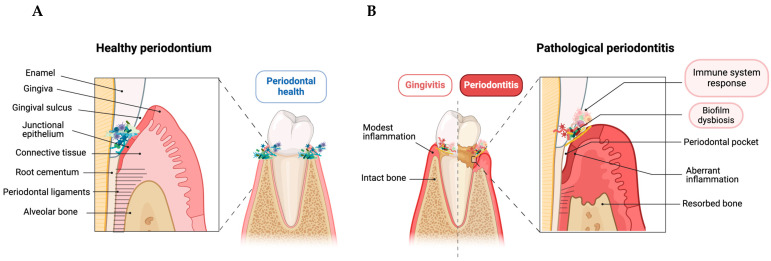
Schematic illustration of the changes in periodontal structure from healthy to pathological periodontitis. (**A**) The healthy periodontium had a complete structure with microbiota homeostasis and a controlled inflammatory environment. (**B**) Pathogenic subgingival microbial biofilms develop and cause host-mediated inflammatory responses without losing alveolar bone, leading to gingivitis. The transition to periodontitis involves an abnormal inflammatory reaction, tooth-supporting structure destruction, periodontal ligament loss, and alveolar bone resorption. This figure was created with BioRender.com.

If biofilm accumulation persists, the inflammatory process becomes irreversible, leading to clinical manifestations of periodontitis involving deeper periodontal tissue disruption that drives the destruction of tooth-supporting structures, loss of periodontal ligaments and cementum attachment, and resorption of the alveolar bone [1,5,11] (Figure 1). In more advanced stages, deepened pocket depths with alveolar bone loss cause tooth mobility, drifting, flaring, and, ultimately, the loss of the affected tooth due to periodontal tissue destruction. A collapse of the bite function is typically the result of advanced cases wherein several teeth are affected [12]. This transition from gingivitis to periodontitis is classified according to clinical manifestations, symptoms, and pathological changes related to molecular, cellular, and immunohistochemical aspects [13,14]. 

The primary etiology of periodontal disease is host-mediated inflammatory and immune responses to microbial plaque accumulation caused by dysbiosis. However, to better understand this disease, focusing on other contributory factors, including host environmental factors, immunological and genetic mechanisms, and environmental host factors like heredity, diet, and lifestyle, is necessary [1,2,15].

Risk factors for periodontitis include daily brushing frequency, routine scaling, smoking, alcohol consumption, heredity, and hypertension [3,15]. Epidemiologically, periodontal disease is linked to comorbidities such as cardiovascular disease, type-2 diabetes, obesity, atherosclerosis, rheumatoid arthritis, osteoporosis, respiratory infections, inflammatory bowel disease, Alzheimer’s disease, nonalcoholic fatty liver disease, chronic kidney disease, and certain cancers [16,17]. Understanding the mechanisms behind this link is crucial for the early identification and prevention of periodontal diseases. 

A dynamic bidirectional interaction between the oral microbiome and the host is essential to establish periodontal diseases. This interaction involves different factors that contribute to the differentiation and maturation of the host mucosa and the development of the host immune system [18,19,20].

The two main aims of this review are: (i) discuss the current knowledge of the immunological mechanisms involved in the initiation and progression of periodontitis, and (ii) give an overview of the different therapeutical interventions in managing periodontitis, focusing the attention on the potential of micro-immunotherapy (MI), an immune regulator-based low dose immunotherapy [21]. Drawing from its applications in various fields, and considering the immunological intricacies of periodontal disease, the aim is to provide insights into how MI may effectively address the complexities of periodontitis based on previous research findings.

## 2. Periodontitis Immunopathology

Dynamic interactions between pathogen products, numerous cell populations, and inflammatory mediators are crucial for homeostasis and inflammatory processes. Thus, it is essential to understand the role of the cells and mechanisms induced in the innate and adaptive immune response by pathogens in periodontitis progression [22,23,24,25,26]. 

### 2.1. Innate Response

**Figure 2:**
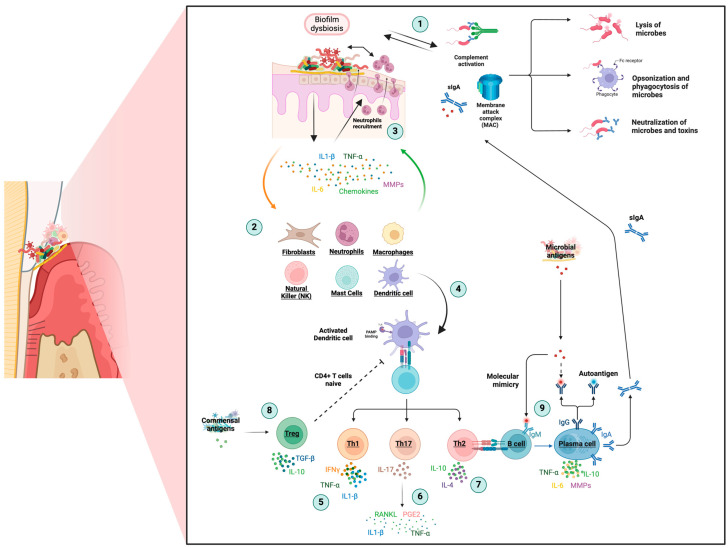
Schematic representation of the immunoregulation in periodontitis progression. Adaptive immune cells activated by an exaggerated innate immune response orchestrate the progression of periodontal inflammation and tissue destruction. The interplay between pathogens, immune cells, and inflammatory mediators in periodontitis immune response is detailed. 1: Lysis of microbial targets through the complement system, secretory IgA (sIgA), and membrane attack complex (MAC); 2: Activation of proinflammatory transcription factors; 3: Migration of neutrophils through the junctional epithelium; 4: Antigen presentation by dendritic cells (DCs) cells; 5: Activation of cellular immunity of Th1 cells by proinflammatory cytokines liberation; 6: IL-17 produced by Th17 exacerbates gingival tissue inflammation and bone loss; 7: Humoral immunity and anti-inflammatory role of Th2 cells; 8: Treg cells role in bone homeostasis; 9: Dual role in antibody production and inflammatory response of B cells. This figure was created with BioRender.com. IL: interleukin; Ig: immunoglobulin; IFN: interferon; MMPs: matrix metalloproteinases; RANKL: receptor activator of nuclear factor kappa-Β ligand; TGF-β: transforming growth factor-beta; Th: T helper; TNF-α: tumor necrosis factor-α. Lines with arrows represent stimulation, while dashed lines indicate inhibition.

The innate immune response is activated when pathogens invade the periodontium, with barriers such as saliva, complement system activity, and the membrane attack complex (MAC) that induce the lysis of microbial targets (Figure 2, point 1). The immune system is activated, amplified, and synchronized through the complement pathway, killing bacteria and activated mast cells, neutrophils, and macrophages [27]. 

However, virulence factors in the biofilm and prolonged inflammation can overcome this well-designed defense system. Periodontal pathogens can hijack and manipulate the host inflammatory response, causing the junctional epithelium to migrate and activate collagen destruction, eventually forming the periodontal pocket [12]. Periodontal pocket refers to a pathologically deepened gingival sulcus around a tooth at the gingival margin, typically because of periodontal disease, allowing for bacterial accumulation and potential damage to the surrounding structures [28]. Therefore, pathogens at the periodontal pocket stimulate the secretion of proinflammatory cytokines as interleukin (IL)-1β, IL-6, IL-23, tumor necrosis factor-alpha (TNF-α), chemokines and antimicrobial peptides by epithelial cells, keratinocytes, fibroblasts, periodontal ligament stem cells (PLSCs), and dendritic cells (DCs) (Figure 2, point 2) [2]. 

Overall, C-X-C motif chemokines (CXCs) tend to activate neutrophils. In contrast, C-C motif chemokines (CCs) are more likely to attract monocytes/macrophages and lymphocytes involved in the polarization of macrophages from an M1 to an M2 phenotype during the transition from acute to chronic inflammation [29]. More specifically, CCL2 and CCL3 are chemotactic for monocytes and lymphocytes; CCL4 is chemotactic for CD4^+^ T cells and is regulated by activated, normal T cell expression and secreted (RANTES); moreover, CCL5 attracts T helper (Th)1 cells, playing a significant role in immune cell migration to sites of periodontal infection [30]. 

As a result of the growing cascade of proinflammatory cytokines and chemokines, neutrophil cells constantly transmigrate through the junctional epithelium to the gingival sulcus, releasing antimicrobial peptides against microbes and stimulating the adhesion and spread of keratinocytes on the tooth surface (Figure 2, point 3) [31]. However, excessive production and secretion of proinflammatory cytokines such as IL-1β, TNF-α, IL-6, and IL-8 by neutrophils can result in the aggravation and expansion of inflammation, making neutrophils fail to kill bacteria of such magnitude and chronic persistence [3,18]. At the same time, this migration into inflamed tissues stimulates the chemotaxis of other non-resident inflammatory cells (macrophages, lymphocytes, plasma cells, and mast cells) to the site of infection. 

Macrophages facilitate bacterial destruction by expressing proinflammatory cytokines such as IL-6 and TNF-α, the secretion of metalloproteinases (MMPs), and collagenases through classically activated M1 macrophages. Activated M2 macrophages secrete many anti-inflammatory factors inhibiting inflammation and promoting tissue regeneration. The ratio of M1/M2 in the gingival tissue and macrophage polarization may be closely related to chronic periodontitis and influenced by microbial populations [31,32]. 

Mast cells migrate to the infection site and undergo explosive degranulation, releasing their granules into the surrounding environment [33]. This process affects endothelial cells and other cell types, causing the production of proinflammatory cytokines and chemokines like TNF-α and RANTES/CCL5, granulocyte-macrophage colony-stimulating factor (GM-CSF), nitric oxide (NO), acid phosphatases, or MMPs [34,35]. In addition, natural killer (NK) cells also contribute to the pathogenesis of periodontitis by binding to oral pathogens and activating the adaptive immune response [2].

Inflammatory mediators in periodontitis contribute to the degradation of the extracellular matrix of the connective tissue. Proteolytic MMP enzymes and their endogenous inhibitors, tissue inhibitors of metalloproteinases (TIMPs), are critical players in tissue destruction. MMP-1, MMP-8, and MMP-13 are involved in alveolar bone destruction by degrading type I collagen, while two gelatinases (MMP-2 and -9) accomplish the degradation of denatured collagen. Furthermore, MMP-9 assists in osteoclast migration, while MMP-13 triggers osteoclast activation, facilitating type I collagen degradation [36,37]. 

This proteolytic response degrades 60–70% of the collagen in the gingival connective tissue, although, gingival tissue repair prevents permanent damage. However, due to host environmental factors, inflammation and tissue damage fail to resolve [38]. Damage leads to pathogen invasion into epithelial tissues and the lamina propria, enhancing tissue breakdown and irreversible periodontal ligament destruction and bone resorption [14,39].

### 2.2. Adaptative Response

The progression of inflammation and tissue destruction is orchestrated by adaptive immune cells, activated by the innate immune response. DC cells within the epithelium recognize microbial antigens through their toll-like receptors (TLRs) and bring them to the lymphoid tissue for antigen presentation to T and B lymphocytes (Figure 2, point 4) [2]. This process involves the release of cytokines such as IL-1β, IL-6, IL-12, and various chemokines [31].

T-helper cytokines are essential in periodontal pathogenesis and disease progression. An intense T lymphocyte stage contributes to cellular immune responses by stimulating various CD4^+^ Th-cell responses [40,41]. Th1 cells activate cellular immunity against intracellular pathogens by proinflammatory cytokines, such as IL-1β, IL-12, IL-2, interferon (IFN)-γ, and TNF-α, associated with periodontal tissue destruction (Figure 2, point 5). IL-17, which is produced by Th17, is also a proinflammatory cytokine with a significant impact, aggravating the inflammation of gingival tissue and the loss of alveolar bone, inducing the expression of the receptor activator of the nuclear factor kappa-Β ligand (RANKL), prostaglandin E2 (PGE2), IL-1β, and TNF-α (Figure 2, point 6) [37].

Th2 cells, which produce cytokines like IL-4, IL-10, and IL-11, play a crucial role in humoral immunity and anti-inflammatory properties, attenuating the destructive host response mediated by mostly Th1 and Th17 (Figure 2, point 7) [38,40,41]. T lymphocytes CD8^+^ regulatory (Treg) cells also play a protective role in alveolar bone homeostasis by suppressing osteoclastogenesis and modulating the innate immune response by attenuating T cells’ excessive inflammation and proliferation (Figure 2, point 8) [2]. Understanding the mechanisms under Th1, Th17, Th2, and Treg subpopulation regulation will improve host-modulatory interventions to resolve inflammation and tissue destruction in periodontal disease.

In periodontitis, B cells, adaptive immune cells, produce antibodies and cytokines [31]. B cells transform into antibody-producing plasma cells that are abundant in inflammatory infiltrates, producing antibodies that facilitate the elimination of bacteria and stop the progression of the disease. The amount and avidity of these antibodies are essential in protecting against periodontitis [3]. B cells can also play a role in immunoregulation and inflammatory response, producing cytokines like TNF-α, IL-6, IL-10, and MMP, which contribute to the degradation of periodontal tissue (Figure 2, point 9) [2].

### 2.3. Osteoimmunology

**Figure 3:**
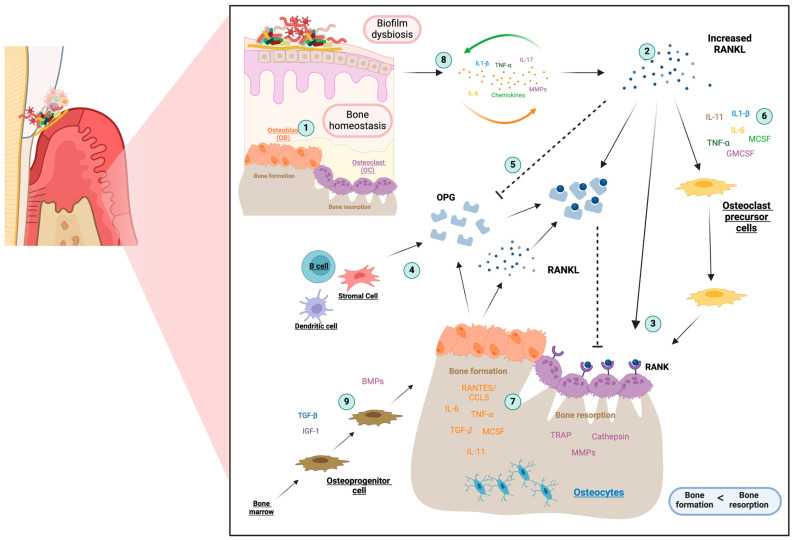
Schematic overview of osteoimmunology in the progression of periodontitis. As a result of amplified immune response and periodontal inflammation reaction, bone homeostasis is disrupted, causing the exaggerated activation of osteoclastogenesis and the destruction of alveolar bone. Different cytokines and chemokines are crucial in bone metabolism, signaling the trafficking of osteoblast (responsible for bone resorption) and osteoclast (responsible for bone formation) precursors. Receptor Activator of Nuclear Factor-kappa B (RANK), its ligand (RANKL), and osteoprotegerin (OPG) are the key regulators involved in periodontitis bone resorption. 1: Bone homeostasis with a dynamic balance between bone formation and resorption; 2: Upregulation of RANKL receptor as a consequence of the exaggerated innate and adaptive immune response; 3: RANKL stimulates resorption activity of mature osteoclasts by binding to RANK; 4: Liberation of the soluble decoy receptor for RANKL, with OPG inhibiting osteoclastogenesis; 5: Increased RANKL liberation inhibited OPG expression, allowing RANKL to interact with RANK; 6: Cascade of proinflammatory cytokines involved in osteoclastogenesis; 7: Role of RANTES/CCL5 as an inflammatory factor essential for communication between osteoclasts and osteoblasts; 8: Factors influencing periodontitis progression such as periodontal pathogens, proinflammatory cytokines, growth factors, MMPs, and RANKL; 9: Role of bone morphogenetic proteins (BMPs) as promoters of osteoblast differentiation, and they enhance bone matrix synthesis and mineralization. This figure was created with BioRender.com. CCL: chemokine (C-C motif) ligand; GMCSF: granulocyte-macrophage colony-stimulating factor; IL: interleukin; IGF: insulin-like growth factor; MCSF: hematopoietic growth factor; MMPs: matrix metalloproteinases; TGF-β: transforming growth factor-beta; TNF-α: tumor necrosis factor-α; TRAP: tartrate-resistant acid phosphatase. Lines with arrows represent stimulation, while dashed lines indicate inhibition.

Periodontal inflammation disrupts the bone remodeling balance, causing the exaggerated activation of osteoclastogenesis and the destruction of alveolar bone, leading to tooth loss [12]. A dynamic balance between bone formation and resorption is necessary for bone remodeling, mediated by osteoblasts and osteoclasts (Figure 3, point 1) [42].

During periodontitis progression, osteoclastogenesis activation is enabled by the upregulation of the RANKL receptor (Figure 3, point 2). RANKL stimulates and maintains the resorption activity of mature osteoclasts by binding its biologically active receptor RANK, expressed on the membrane of osteoclast precursors and mature osteoclasts (Figure 3, point 3) [43,44]. However, a soluble decoy receptor for RANKL, osteoprotegerin (OPG), inhibits osteoclastogenesis (Figure 3, point 4) [43]. Inhibiting OPG expression allows RANKL to interact with its receptor RANK to facilitate bone degradation and inhibit osteoblasts’ differentiation, decreasing osteocalcin production and new bone formation (Figure 3, point 5) [2,45].

RANKL-dependent osteoclastogenesis may be initiated by contact with pathogenic factors and interaction with TLR and maintained by an overactivated immune response, infiltration of immune cells, and overproduction of proinflammatory cytokines. A cascade of inflammatory proteins and enzymes is directly involved in periodontitis’ osteoclastogenesis, including proinflammatory cytokines such as IL-1β, IL-6, IL-11, IL-17, and TNF-α (Figure 3, point 6) [40,43,44].

Chemokines are crucial in bone metabolism, signaling the trafficking of osteoblast and osteoclast precursors [30,46]. They participate in normal bone turnover but may exacerbate periodontal disease severity. Pathological increases in CCL4, RANTES/CCL5, or CCL7 release from activated osteoblasts may promote local bone resorption by stimulating chemotactic recruitment and RANKL differentiation of pre-osteoclasts (Figure 3, point 7) [47]. RANTES/CCL5 is an inflammatory factor essential for communication between osteoclasts and osteoblasts [48]. Inflammatory/osteoclastogenic cytokines, such as TNF-α and IL-1β, can stimulate osteoclastic activity and elevate calcium levels in the bone. When exposed to these signals, osteoblasts secrete RANTES, which act as a chemokine for preosteoclasts, promoting their migration to the site of future bone resorption [46,48].

Bone loss associated with periodontitis may be the indirect result of the exacerbated T-cell response and cellular interactions in the osteoimmune system, dependent on the balance between positive and negative factors expressed by T cells. Periodontal disease progression results from a combination of factors, including periodontal pathogens, high levels of proinflammatory cytokines, growth factors, MMPs, and RANKL (Figure 3, point 8).

A central role seems to be played by two cytokines in periodontitis: TNF-α and IL-1β. Several cell types synthesize these two cytokines in periodontal tissue, stimulating the release of numerous mediators, including IL-6, IL-8, MMPs, PGE2, and RANKL [40,42,49]. Bone morphogenetic proteins (BMPs) are a subgroup of transforming growth factor-beta (TGF-β) known for their osteoinductive potential in bone remodeling [50,51]. They promote osteoblast differentiation and enhance bone matrix synthesis and mineralization (Figure 3, point 9) [52]. BMPs indirectly affect bone homeostasis and immune cell differentiation, activation, and immunoregulatory function, highlighting their role in both anabolic and catabolic aspects of bone remodeling [53].

Ultimately, periodontitis is a complex disease with a nonlinear pattern that affects the immune system differently. Despite progress in understanding immune cell function, much remains unknown about how cells interact regarding the pathogenesis of periodontal disease. For future management of periodontal disease, it is necessary to identify the critical interaction between these numerous factors involved in the inflammatory process and periodontal tissue destruction for an integrated view of its pathogenesis [38].

## 3. Approaches in Periodontal Therapy 

### 3.1. Strategies in Periodontal Treatment 

**Figure 4:**
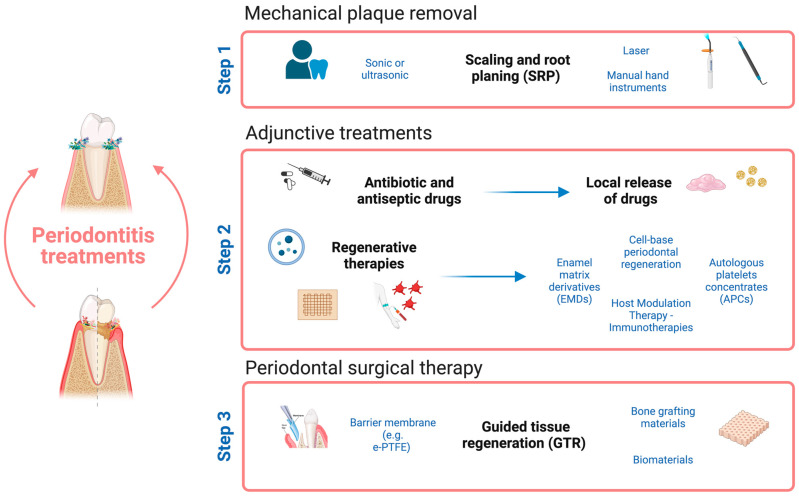
Illustration of the diverse periodontitis treatment approaches available, following graduated steps. The first-line approach in therapy is mechanical plaque removal (Step 1)—removing supragingival dental biofilm—followed by adjunctive therapies. Adjunctive therapies may include antibiotic and antiseptic drugs—by local or systemic release—or regenerative therapies targeting inflammation and regeneration of periodontal tissue lost (Step 2). In more advanced cases, periodontal surgical therapy may be necessary to restore gums and supporting tissues, helped by various biomaterials applied in guided tissue regeneration (GTR) (Step 3). This figure was created with BioRender.com.

The treatment for periodontitis involves eliminating infection, reducing inflammation, and regenerating bone and soft tissue to anchor teeth. Different treatment approaches are used to prevent and treat the disease with combined therapies, depending on the stage of the disease (Figure 4) [54,55,56,57]. The first step is based on mechanical plaque removal, and behavior change, scaling, and root planing are the gold standard (Figure 4, step 1) [57]. Successful periodontal therapy involves the use of adjunctive treatments in addition to mechanical interventions focused on reducing inflammation, inhibiting bacterial growth, and promoting tissue repair (Figure 4, step 2). The last step in periodontal therapy involves surgical interventions like guided tissue regeneration (GTR), which uses barrier membranes and other biomaterials to guide new tissue growth. This treatment can enhance angiogenesis promotion, alveolar bone regeneration, or cementum regeneration (Figure 4, step 3) [19,58,59]. 

Adjunctive periodontitis therapy has evolved over time to immunoregulation therapies, considering the role of inflammatory responses and immune dysregulation [31]. Various immunomodulators and host modulatory agents are being developed to improve treatment [60,61]. Regenerative therapies based on autologous platelet-rich fibrin (PRF) [62,63,64], concentrated growth factors (CGFs) [65,66], or enamel matrix derivatives (EMDs) [67] are used to accelerate healing and regenerate periodontal tissue. Non-steroidal anti-inflammatory drugs (NSAIDs) [68], tetracycline [69,70], bisphosphonates, growth factors, and anti-inflammatory drugs, such as anti-cytokines targeting TNF-α, IL-1, IL-6, T and B cells, or TLR inhibitors [60,61], are also available but not practical for periodontal treatment due to high costs and potential side effects [71]. 

Therefore, it is crucial to develop effective treatment strategies that consider the factors contributing to the immunoinflammatory response in periodontal diseases and develop potential drug targets based on the key molecules involved in periodontitis’ innate and adaptive immune systems. In addition, further research on immunoregulation in periodontitis treatment is needed to address these issues and develop more effective and safe treatments. 

### 3.2. Micro-Immunotherapy Therapeutic Landscape and Its Potential in Periodontitis 

Within the framework of adjuvant therapies in periodontitis, immunomodulatory strategies offer a wide range of possibilities for periodontitis treatment. Immunomodulatory strategies may be directed to explicitly targeting the elements that articulate the immune-inflammatory response involved in the onset and progression of the disease.

As aforementioned, there is an existing network of signaling molecules involved in the activation and development of immune responses, and the crosstalk within this network has an enormous impact on the regulation of cellular and tissue functions [72,73,74]. Moreover, signaling molecules play essential roles in cell–cell communication under both physiological and pathological processes, and maintaining or recovering these signaling molecules’ homeostatic equilibrium is fundamental to preventing and/or treating, for example, inflammatory, allergic, and autoimmune diseases [75]. 

In this regard, the low/ultra-low dose-based approach of micro-immunotherapy (MI) constitutes a high-potential approach within the framework of immunomodulation in periodontitis. Micro-immunotherapy medicines (MIMs) mainly focus on maintaining and restoring homeostasis using immune signaling molecules, such as cytokines, growth factors, hormones, neurotransmitters, and/or nucleic acids, prepared in low doses (LDs) or ultra-low doses (ULD) [76,77]. Within MIM, the quantity of these active ingredients, expressed through centesimal Hahnemannian (CH) dilutions, directs biological responses towards either activation or inhibition, contingent upon the CH range utilized [78]. One of the particularities of this approach resides in lowering the concentration of these immune messengers to overcome the main limitations linked to the traditional use of these molecules [79,80,81]. MIM aims to maintain a therapeutic effect while improving treatment tolerability and reducing side effects. MI formulations either encompass a sole active substance or a distinctive combination of several ingredients in order to exert a multi-target approach that aims to modulate multiple targets simultaneously [77]. MIMs are manufactured in the form of homeopathic medicinal products consisting of sugar-based pillules impregnated with the corresponding active substances and prepared according to a serial dilution 1:100 process and a kinetic agitation–Serial Kinetic Process (SKP) [82]. They are intended for oromucosal administration, to be administered in a sequential manner, in sequences of five or ten different capsules (MIM-1 on Day 1, MIM-2 on Day 2, and so forth), to provide a unique combination of active substances in chronological order [83,84,85]. Sublingually administered in the oral cavity, this therapy is interesting as an adjunct to supportive periodontal therapy as well as a preventive treatment due to its proximity to gingival tissues, which allows it to act both locally and systemically. The medication dosage is determined by the patient’s condition’s characteristics and severity, which are evaluated by the physician and influence the type and dosage prescribed.

This therapeutic approach is based on initiating and/or regulating immunoregulatory responses [86]. Some studies have proposed that even in low-dose preparations, active ingredients could act by activating cellular or plasmatic receptors [81]. This is achieved through cascades of amplification mechanisms, with few molecules per cell needed [87,88]. Many biological systems are sensitive to ultra-low ligand concentrations, including immune responses, hormonal functions, enzyme activities, and cyclic adenosine monophosphate (cAMP) production [89,90,91,92]. Some studies suggest that the mechanism of action of these LDs of signaling molecules is related to the phenomenon of hormesis, an adaptive response characterized by an inverse J-shaped dose–response curve [77,93,94,95,96]. Examples of cellular signaling molecules displaying a hormetic response include growth factor signaling pathways or cytokines like TNF or IL-6 [97,98]. 

Several studies show encouraging results of the efficacy of low-dose medicine employing signaling molecules, such as cytokines at low doses in inflammatory diseases. Similar to MI, it employs low doses of signaling molecules such as interleukins. For instance, LD IL-10 and anti-IL1-α antibodies demonstrate promise in colitis [99] and osteoarthritis models [100], while a combination of IL-4, IL-10, and anti-IL1-α antibodies outperforms DMARDs in rheumatoid arthritis [101]. LD progesterone and IL-10 also exhibit potential in endometriosis treatment, suggesting advancements in targeted immunomodulation [102]. This therapy, referred to as Low Dose Medicine (LDM), originates from the concept of psycho-neuro-endocrine-immunology, consisting of the existing crosstalk between the immune system and the psycho-neuro-endocrine systems [74].

Regarding the pleiotropic effects of the cytokines and their implication in a broad spectrum of chronic systemic immune-mediated diseases, MI could be used in various disorders, neurological disorders [78,103], infectious diseases [21,104], disorders of the bones and joints [77,105], autoimmune diseases [76], allergies [106], and as a complementary treatment for oncology [83]. 

In recent years, there has also been a notable increase in the number of studies and scientific articles dedicated to MIM. This growing attention is reflected in the research delving into both the mode of action and the effectiveness of MIM as an immunoregulatory treatment, either conducted in vitro or in vivo, and referring to unitary medicines with sole active ingredients [78,88,107,108,109] or complex MI formulations [21,76,77,78,82,83,85,103,104,105,106]. Particularly noteworthy are investigations that delve into the study of MIM applied in the field of inflammatory diseases [77,82,105,106]. 

An example of MIM application in treating inflammatory diseases is the sequential medicine 2LARTH^®^. This medication, containing ultra-low doses (ULDs) of cytokines TNF-α, IL-1β, and IL-2 along with other immune factors, has already demonstrated anti-inflammatory effects in vitro, in human peripheral enriched monocytes, by reducing the levels of proinflammatory cytokines such as TNF-α, IL1-β, and IL-6 in a recent publication of 2018 [82]. In a collagen-induced arthritis (CIA) murine model of rheumatoid arthritis (RA), this medicine also exhibited a reduction in arthritis clinical signs and decreased systemic TNF-α levels [105]. The strategy and therapeutic targets of 2LARTH^®^ in RA pathogenesis have been equally recently reviewed [77]. In parallel with this, another in vitro study also showed the anti-inflammatory effects of TNF-α and IL-1β prepared at ULDs in reducing TNF-α secretion in human primary monocytes and THP-1 cells [88].

Hence, considering that, on one hand, the results of various MIMs in several therapeutic fields, particularly in inflammation control [77,106] and in modulating immune cell function [104,107], are crucial dysregulated aspects in periodontitis, and that, on the other hand, current treatments or preventive solutions for periodontitis are scarce and still limited due to the side effects and routes of administration of existing therapies [60,61,70,71], the use of MIM treatments emerges as a highly interesting therapeutic strategy for periodontitis treatment.

Thus, in the context of periodontitis, several studies with MIM have been set to explore the impact of various proteins, such as BMPs in LDs and ULDs, either in the form of single MIM formulations or a combination of immune mediators [84,108]. From their characteristics, these treatments may have the potential to explicitly target the elements that articulate the immune-inflammatory response involved in disease onset and progression, but not exclusively, as they might conceivably be useful in promoting the regeneration of soft tissues, as tissue loss is distinctive of periodontitis progression.

The first study published in the Journal of Periodontology in 2021 was set to evaluate the beneficial effect of BMPs in LDs for periodontal regeneration and repair [108]. In this research, a MIM consisting of BMP4 in LD presented anti-inflammatory properties when used in human gingival fibroblasts (hGFs) in culture. The study also highlighted the impact of the medicine on collagen metabolism in the same cell. In addition, within a more complex 3D model, LD BMP4 recovered tissue viability under inflammatory conditions, thus concluding a potential long-term effect of the MIMs promising approach for treating periodontitis.

In line with this study, the effect of a more complex formula with a specific combination of immune mediators was also evaluated [84]. In hGF culture, anti-inflammatory properties and positive impacts on collagen metabolism were also confirmed and, in the 3D model of oral mucosa, this sequential complex formula increased collagen content. Such elevation in collagen levels could be beneficial as it promotes the regeneration of gingival tissue and restoration of periodontal health, thereby enhancing the function and stability of the affected teeth. The active substances of this sequential complex formula share the presence of LD BMP-2 and BMP-4 among other substances such as the cytokines IL-1β, IL-6, and TNF-α in ULD or TGF-β in LD. These results manifest the opportunity of combining different active ingredients with different specific potential targets, each related to periodontitis immunopathology, like substances that can help decrease and resolve inflammation or substances acting on the extracellular matrix. 

Therefore, these results demonstrate anti-inflammatory and regenerative properties in two in vitro soft-tissue models of periodontitis. As mentioned, sustained inflammation in the gingival tissues results in collagen degradation, contributing to the loss of structural and functional support for the teeth. In this regard, MIM based on BMPs and cytokines at LD and ULD has demonstrated anti-inflammatory properties that could modulate the proteolytic response of the collagen and may benefit soft tissue healing. Nevertheless, the specific mechanism of action for this MIM still needs to be thoroughly evaluated. 

In practical terms, combining available strategies, such as mechanical or surgical treatments, with the MI approach could be an effective and comprehensive strategy to address the complexity of periodontitis. The synergy between treatments may maximize benefits for the regeneration of affected tissues. However, it is crucial to conduct additional studies that explore the efficacy of this combined approach. 

Lastly, it should be mentioned that another cytokine involved in periodontitis immunopathology is RANTES/CCL5. This cytokine is responsible for regulating the migration and activation of various immune cells, including T cells, monocytes, and dendritic cells, within inflamed tissues [110,111]. Under these considerations, a MIM consisting of ULD of RANTES may have the potential to modulate chemokines overexpression, as well as be involved in bone resorption and the overproduction of proinflammatory cytokines [109]. 

A preliminary follow-up study treated patients suffering from systemic immune-mediated diseases (SIDs) who underwent dental surgery for fatty-degenerative osteolysis jawbone (FDOJ) cavitations with unitary ULD RANTES MIM treatments. These patients showed altered CCL5 levels, implicated in forming inflammatory infiltrates [112]. Although a larger cohort of patients and placebo controls are still necessary, this study showed a reduction in RANTES levels for the patients treated with MIM [109]. For a more thorough understanding of ULD RANTES MIM’s specific interaction in periodontitis, further comprehensive testing is needed.

**Figure 5:**
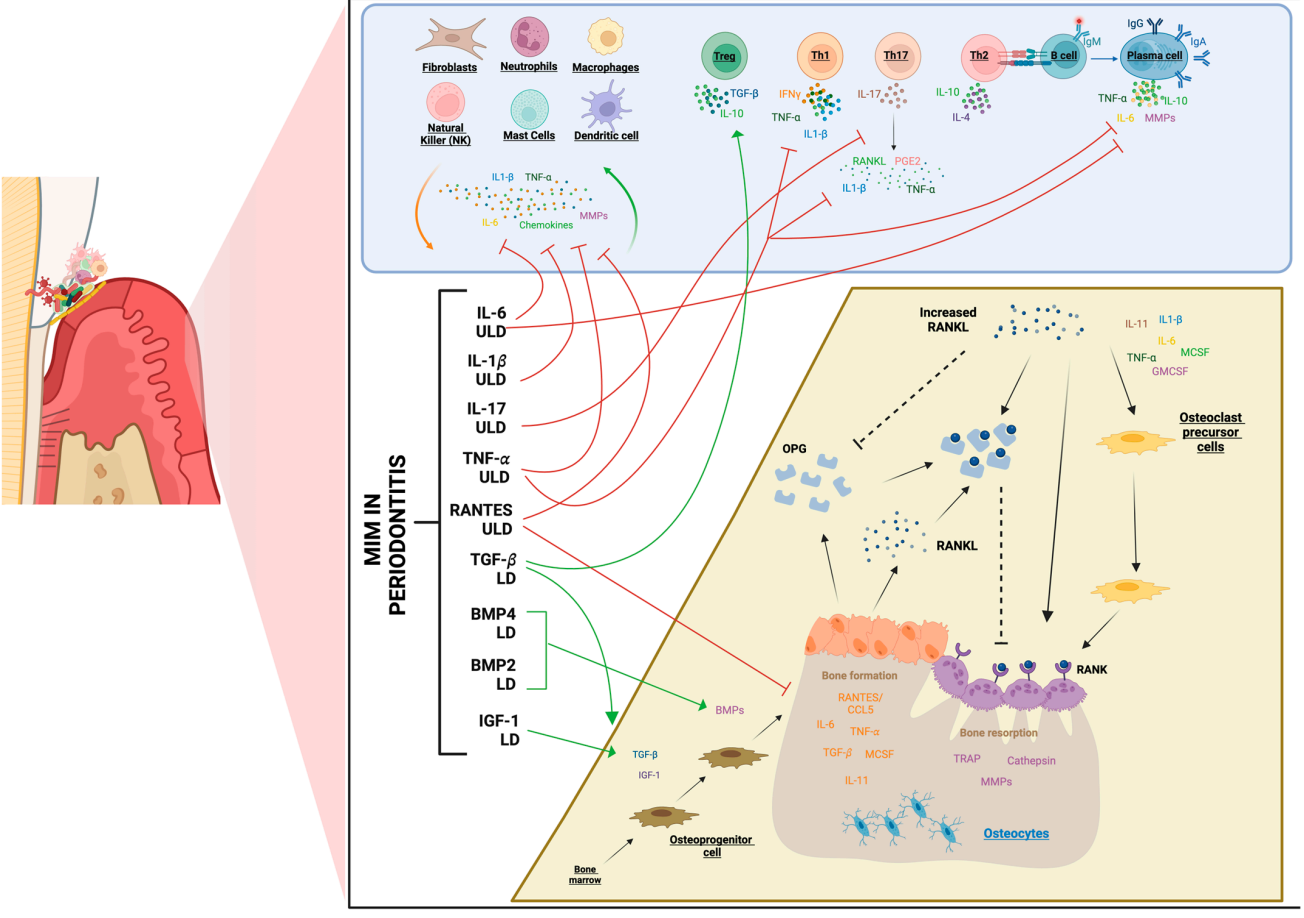
Exploring low-dose (LD) and ultra-low dose (ULD) immunoregulators and their role in immunopathology periodontitis. Representation of the immunopathological processes underlying periodontitis and the potential of micro-immunotherapy medicines (MIMs) in areas that could intervene as an adjunct and novel therapeutic approach for treating periodontitis. Red arrows could minimize the cascade of proinflammatory cytokines involved in the progression of periodontitis. Green arrows could stimulate the regenerative and osteoinductive potential of immunoregulators. This figure was created with BioRender.com. BMP: bone morphogenetic protein; CCL: chemokine (C-C motif) ligand; GMCSF: granulocyte-macrophage colony-stimulating factor; IGF: insulin-like growth factor; IL: interleukin; IFN-γ: interferon gamma; MCSF: hematopoietic growth factor; PGE2: prostaglandin E2; RANK: receptor activator of nuclear factor kappa-Β; RANKL: receptor activator of nuclear factor kappa-Β ligand; TGF-β: transforming growth factor-beta; Th: T helper; TIMP-1: tissue inhibitor of metalloproteinases-1; TNF-α: tumor necrosis factor-α; TRAP: Translating Ribosome Affinity Purification; RANTES: regulated on activation, normal T cell expressed and secreted. Lines with arrows and green lines represent stimulation, while dashed lines and red lines indicate inhibition.

As a summary, Figure 5 schematically and graphically presents various immunomodulators employed in MIM, both in LD and/or ULD, that have been studied for the treatment of periodontitis. These active substances have been investigated both individually and/or as integral components of more complex formulations, exploring their potential role in the immunoregulation of this condition.

The studies shared in this review demonstrate promising results, especially considering that periodontitis is intricately linked to immunopathology. These findings suggest that immunomodulatory interventions hold significant promise in the management of periodontitis, potentially offering novel therapeutic avenues for addressing the immunological aspects of this condition.

## 4. Conclusions and Future Directions

This review focuses on the main etiopathogenetic mechanisms involved in periodontitis and on the currently available and developing therapeutic solutions for those patients suffering from this gingival inflammatory-mediated disease. The second part of the review discusses more deeply the potential of MI as a valuable therapeutic solution for periodontal health management. As highlighted, MI emerges as a versatile and promising field with implications for diverse health conditions. A wide range of preclinical and clinical studies are presented to demonstrate the effectiveness of immunotherapy in LD, suggesting a multifaceted role for MI. Studies concerning the potential of MI in the context of inflammatory-related diseases, allergy inflammation diseases, immune cell modulation, viral infections, and dermatological conditions are presented. 

Now, the current evidence on MI allows us to explore its potential in the context of periodontitis as a tool to target those pathways involved in the progression of the disease (Figure 5). Immune mediators used in LD or ULD like IL-1β, TNF-α, IL-6, or chemokines RANTES/CCL5 are implicated in the activation of neutrophil migration and stimulation of non-resident inflammatory cells, responsible for the growing cascade of proinflammatory cytokines leading to tissue destruction. On the other hand, the employment of LD and/or ULD of cytokines such as IL-4, IL-12, and interferon-gamma (INF-γ), which presented T-cell stimulatory capacity and the modulation of T-cell subpopulations, could also be suitable therapeutic targets related to the adaptive response involved in the progression of periodontitis. In relation to the process of bone remodeling, several cytokines, such as BMPs, RANTES, IL-1β, IL-6, IL-11, or TNF-α, are employed at LD or ULD in MI formulas; thus, they can offer therapeutic benefits in balancing the process during periodontitis progression towards optimal bone regeneration and tissue repair.

Finally, collagen metabolism also plays an important role in the maintenance of the extracellular matrix to prevent damage to gingiva connective tissue. Therefore, it is important to highlight the studies with LD immune mediators that have shown the prevention of collagen destruction and improved tissue viability. At the same time, these LD and ULD-based treatments also demonstrate anti-inflammatory properties that contribute to the prevention of tissue damage. These studies are the first one that relates the use of LD immune mediators and the treatment of periodontitis. 

While our study offers valuable insights into the potential of MI in managing periodontitis, it is important to recognize certain limitations inherent in the current body of clinical evidence and follow-up procedures. The review draws upon available clinical trials and studies on MI; however, the relatively limited number of these resources could suggest areas for further exploration and research. Furthermore, the relatively short follow-up duration in some studies presents an opportunity for future investigations to delve deeper into the long-term effects and sustainability of MI in periodontitis management.

Therefore, although it is necessary to fully understand LD immunomodulatory and immunostimulant capacity in the context of periodontal disease with further research, the incorporation of LD immunotherapy as an adjunct to periodontal therapy introduces a novel approach to addressing periodontitis. In conclusion, MI emerges as a versatile and promising field with implications for diverse health conditions. The findings presented in this exploration offer valuable insights into the potential applications of MI in the periodontal disease context. As the field advances, continued research in preclinical and clinical trials is imperative to solidify the evidence base, ensuring the safe and effective integration of MI into clinical practice.

## Abbreviations

BMP: bone morphogenetic protein; cAMP: cyclic adenosine monophosphate; CC: C-C motif chemokine; CH: centesimal Hahnemannian; CGFs: concentrated growth factors; CXCL: C-X-C motif chemokines; DCs: dendritic cells; EMD: enamel matrix derivate; FDOJ: fatty-degenerative osteolysis jawbone; GMCSF: granulocyte macrophage colony-stimulating factor; GTR: guided tissue regeneration; hGFs: human gingival fibroblasts; IGF: insulin-like growth factor; IL: interleukin; IFN: interferon; LD: low dose; LPS: lipopolysaccharide; MAC: membrane attack complex; MCSF: hematopoietic growth factor; MI: micro-immunotherapy; MIM: micro-immunotherapy medicine; MMPs: matrix metalloproteinases; NSAIDs: non-steroidal anti-inflammatory drugs; OPG: osteoprotegerin; PDGF: platelet-derived growth factor; PGE2: prostaglandin E2; PLSC: periodontal ligament stem cells; PRF: platelet-rich fibrin; RANK: Receptor activator of nuclear factor kappa-Β; RANKL: receptor activator of nuclear factor kappa-Β ligand; SIDs: systemic immune-mediated diseases; TGF-β: transforming growth factor-beta; Th: T helper; TIMP-1: tissue inhibitor of metalloproteinases-1; TLRs: toll-like receptor; TNF-α: tumor necrosis factor-α; TRAP: Translating Ribosome Affinity Purification; Treg: T lymphocytes CD8+ regulatory; RANTES: regulated on activation, normal T cell expressed and secreted; sIgA: secretory IgA sIgA; Treg: T lymphocytes CD8+ regulatory; ULD: ultra-low doses.
